# Targeting acetyl-CoA carboxylase 1 for cancer therapy

**DOI:** 10.3389/fphar.2023.1129010

**Published:** 2023-02-10

**Authors:** Yong Yu, Qingzhu Nie, Ziyi Wang, Yu Di, Xiaolong Chen, Kaiming Ren

**Affiliations:** ^1^ Department of Ophthalmology, Shengjing Hospital of China Medical University, Shenyang, Liaoning, China; ^2^ Department of Thoracic Surgery, First Hospital of China Medical University, Shenyang, Liaoning, China; ^3^ Department of Thoracic Surgery, Shengjing Hospital of China Medical University, Shenyang, Liaoning, China

**Keywords:** metabolism, cancer, acetyl-CoA carboxylase 1, fatty acid, fatty acid synthesis

## Abstract

Metabolic adaptation is an emerging hallmark of tumors. *De novo* fatty acid synthesis is an important metabolic process to produce metabolic intermediates for energy storage, biosynthesis of membrane lipids and generation of signaling molecules. Acetyl-CoA carboxylase 1 (ACC1) is a critical enzyme in the fatty acid synthesis, which carboxylates acetyl-CoA carboxylic acid to form malonyl-CoA. The role of acetyl-CoA carboxylase 1 in fatty acid synthesis makes it a promising therapeutic target for various metabolic diseases such as non-alcoholic fatty liver disease, obesity and diabetes. Tumors have a high energy flow and a strong dependence on fatty acid synthesis. Thus, acetyl-CoA carboxylase inhibition has become a potential choice for anti-tumor therapy. In this review, we first introduced the structure and expression pattern of Acetyl-CoA carboxylase 1. We also discussed the molecular mechanisms of acetyl-CoA carboxylase 1 in the initiation and progression of various cancer types. Furthermore, acetyl-CoA carboxylase1 inhibitors has also been discussed. Collectively, we summarized the interplay between acetyl-CoA carboxylase 1 and tumorigenesis, indicating acetyl-CoA carboxylase 1 as a promising therapeutic target for tumor management.

## Introduction

Fatty acid metabolism, particularly the synthesis of fatty acids, is an essential metabolic process that produces various metabolic intermediates for energy storage, biosynthesis of membrane lipids and production of signaling molecules (Snaebjornsson et al., 2020). Thus, fatty acid synthesis, uptake, and oxidation contributes to the maintenance of biological function, and blocking these processes regulating fatty acid metabolism may affect cell survival. Alterations in fatty acid metabolism are one of the most significant metabolic changes involved in tumorigenesis. Increased fatty acid synthesis supports tumor cell survival and growth (Munir et al., 2019; Broadfield et al., 2021). Therefore, targeting fatty acid metabolism, particularly fatty acid synthesis, is gradually considered as a promising anti-tumor therapeutic strategy.

**FIGURE 1:**
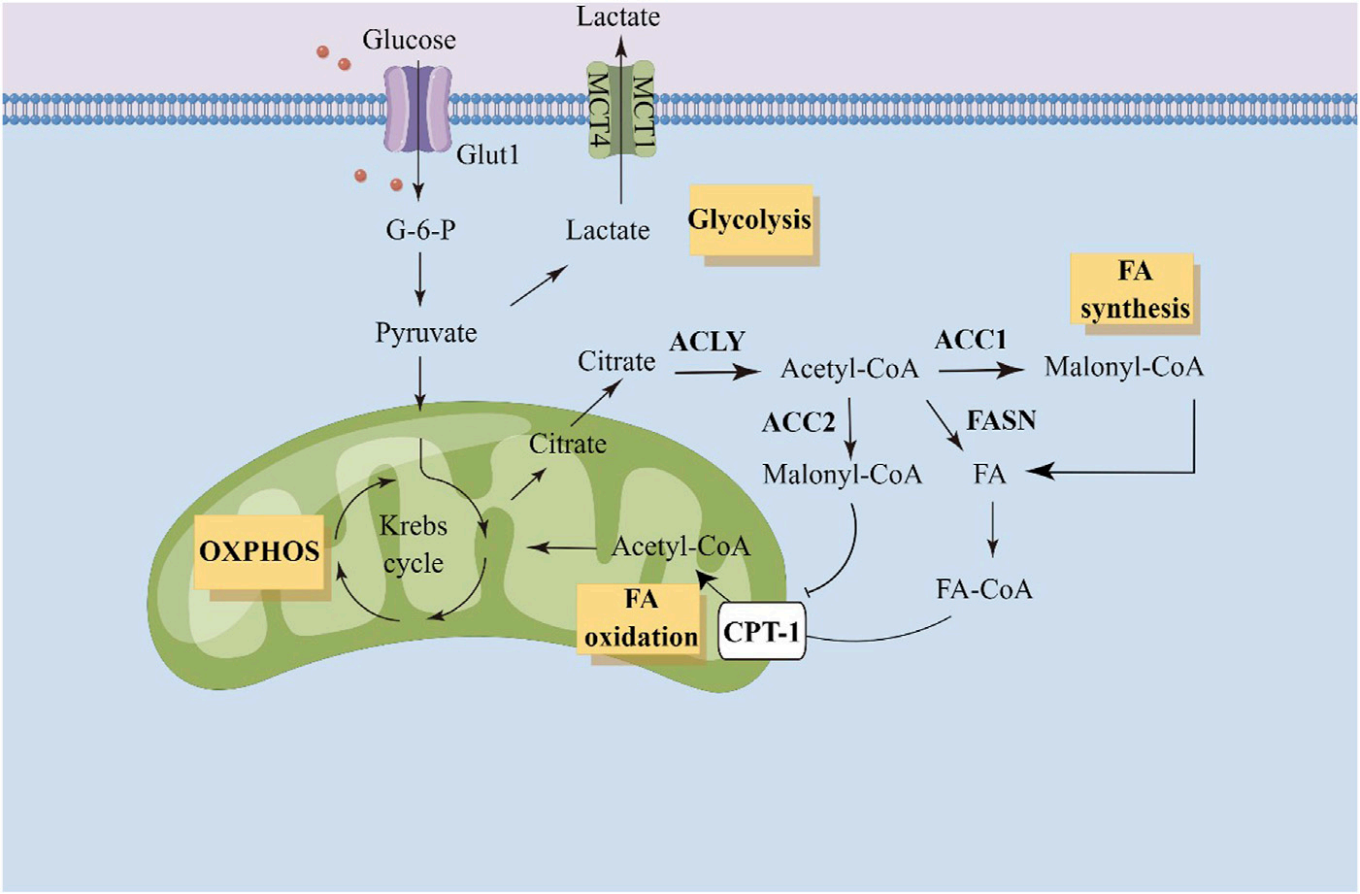
Illustration of fatty acid metabolism in tumor cells. ACC, acetyl-CoA carboxylase; ACLY, ATP citrate lyase; Glut1, gluose transporter 1; G-6-P, Glucose-6-phosphate; OXPHOS, oxidative phosphorylation; FA, fatty acid; CPT-1, carnitine palmitoyltransferase 1.

Glucose metabolism interacts with fatty acid metabolism at the point of citric acid, an intermediate of the tricarboxylic acid cycle. Converting carbon from citrate to bioactive fatty acids requires several steps. These steps involve ATP citrate lyase (ACLY), acetyl-CoA carboxylase (ACC), fatty acid synthase (FASN), and acyl-CoA synthase (ACSL) (Brownsey et al., 2006; Zaidi et al., 2012; Röhrig and Schulze, 2016). ACC carboxylates acetyl-CoA to form malonyl-CoA, and is a critical enzyme in the fatty acid synthesis. Two isoforms of ACC exist in eukaryotic cells (Kim, 1997). ACC1 is highly enriched in adipogenic tissues and ACC2 exists in oxidized tissues. Since ACC1 and ACC2 are found in different tissues, they have distinct metabolic roles. The first rate-limiting step in fatty acid synthesis is mediated by ACC1, which converts acetyl-CoA to malonyl-CoA in the cytoplasm. Malonyl-CoA is consumed by FASN for *de novo* fatty acid synthesis, while consuming large amounts of NADPH. ACC2 localizes to the outer mitochondrial membrane. Malonyl-CoA produced by ACC2 inhibits carnitine palmitoyltransferase 1 (CPT-1) and prevents fatty acids entering mitochondria to drive fatty acid oxidation. Malonyl-CoA synthesized by ACC1 is considered as a substrate for fatty acid synthesis, while malonyl-CoA synthesized by ACC2 primarily participates in the inhibition of CPT-1 and fatty acid degradation (Wang et al., 2022). The overview of fatty acid metabolism has been shown in Figure 1.

## Structure and regulation of human acetyl-CoA carboxylase 1

The critical function of ACC1 in fatty acid metabolism has sparked huge amounts of investigations on its structure. The structure of eukaryotic ACC1 is polypeptide, containing main domains including biotin carboxylase (BC), biotin containing carboxyl carrier protein (BCCP), carboxyltransferase (CT), as well as an interaction domain (BT) and a non-catalytic center domain (CD) (Tong, 2012). CD is composed of four domains: N-terminal CDN, the linking CDL, and the tandem C-terminal CDC1 and CDC2 (Hunkeler et al., 2016). Acetyl-CoA carboxylation is a two-step reaction: BCCP linked-biotin is partially carboxylated, and ATP is consumed by BC domain; Next, the generated carboxybiotin is transported to the CT domain, and the carboxyl group is exchanged to acetyl-CoA (Hunkeler et al., 2018).

It has been shown that ACC1 is regulated by protein phosphorylation, allosteric regulator binding and protein-protein interactions. Human ACC1 is inactivated by AMPK phosphorylation and cAMP-dependent protein kinase (PKA) phosphorylation (Ha et al., 1994). Inhibition of ACC1 phosphorylation by AMPK is critical for controlling fatty acid metabolism and cellular response to metabolic stress. AMPK is an essential regulator of metabolism and is activated by both AMP-dependent and AMP-independent mechanisms (Fullerton et al., 2013). Mice with knock-in mutations in both ACC1 (Ser79) and ACC2 (Ser212) exhibit increased fatty acid synthesis and inhibition of fatty acid oxidation compared with wild-type mice (Fullerton et al., 2013). Moreover, inhibition of ACC1 by AMPK is essential for maintaining NADPH levels under conditions of energy stress, which is achieved by reducing NADPH consumption in fatty acid synthesis and enhancing NADPH produced by fatty acid oxidation. ACC1 inhibition compensates AMPK activation and promotes anchor-independent growth and solid tumor formation *in vivo*, whereas ACC1 activation attenuates these processes (Fullerton et al., 2013).

In addition, the tumor suppressor BRCA1 is speculated to regulate ACC1 (Shen and Tong, 2008). BRCA1 interacts with ACC1 and participates in the regulation of lipogenesis in adipose tissues. BRCA1 binds to ACC1 through its C-terminal tandem BRCT domain, which recognizes phosphorylated Ser1263 in the CD region of ACC1 (Hunkeler et al., 2018). Binding to BRCA1 can prevent the dephosphorylation of Ser80, thereby inhibiting the activation of ACC1. Mutations in the BRCT domain could disrupt the binding of BRCA1 to ACC1 and lead to elevated adipogenesis, which is critical for tumor growth. The interaction between cytosolic ACC1 and BRCT domain of BRCA1 is mainly localized in the nucleus, but also occurs in the cytosols. The interaction between the dimerized BRCT domain and the ACC1 phosphorylation loop triggers the polymerization of the extended ACC1 dimer into an ACC1-BRCT double stranded filament with a unique CD conformation and a monomeric BC domain (Fullerton et al., 2013). Post-translational modifications of ACC1 contribute to the function of ACC1, which may provide promising therapeutic targets for anti-tumor strategies.

## Acetyl-CoA carboxylase 1 and malignancies

**FIGURE 2:**
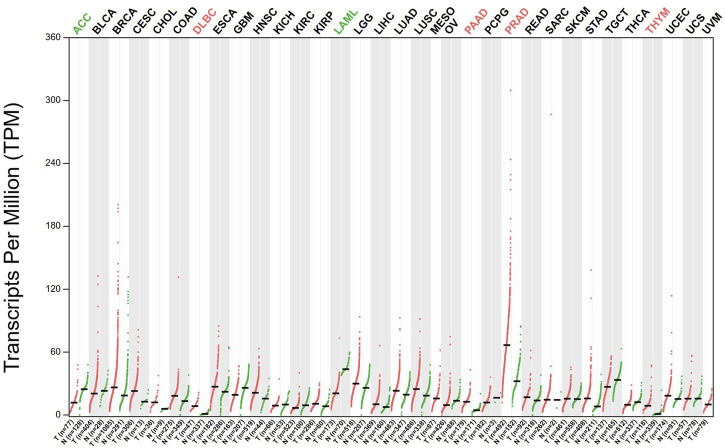
The expression levels of ACC1 in different cancer types have been analyzed by GEPIA database.

ACC1 has been implicated in the initiation and development of multiple malignancies. The expression levels of ACC1 have been analyzed by GEPIA database and illustrated in Figure 2. In this part, we discussed the complex and dynamic role of ACC1 in diverse cancer types.

## Acetyl-CoA carboxylase 1 and acute myeloid leukemia

Acute myeloid leukemia (AML) is a malignant tumor of myeloid stem cell precursors (Prada-Arismendy et al., 2017). ACC1 functions as a substrate for the TRIB1-COP1 complex, and its degradation is important for mediating metabolic adaptations to provide an energetic advantage to support tumor development (Ito et al., 2021). It has been shown that ACC1 binds to Tribbles through the α-helical region of its N-terminal BC domain. ACC1 stabilization impairs Trib1-COP1-induced leukemia progression by increasing ROS levels and NADPH depletion. Thus, ACC1 stabilization abrogates the self-renewal capacity of leukemic stem cells and promotes myeloid differentiation in mouse models of AML. Therefore, small molecule inhibitors targeting the ACC1-helical region are promising for AML treatment. A drug combination redeployed with bezafibrate and medroxyprogesterone acetate (designated BaP) exhibits anti-tumor activity in AML cells (Southam et al., 2015). Mass spectroscopy-based lipidomics and gene expression levels of lipogenic enzymes has indicated that ACC1, FASN, and stearoyl-CoA desaturase 1 (SCD1) were changed in AML cell lines treated with BaP (Southam et al., 2015). Supplementation with oleate, an enzyme product of SCD1, protected AML cells from BaP-induced cell death and reduced the level of BaP-induced ROS. Precious studies indicated that monounsaturated fatty acids contribute to the tumor initiation and development (Scott et al., 2022). Mechanistically, BaP plays an anticancer role by disrupting monounsaturated fatty acid synthesis. BaP has been used in clinical trials to treat leukemia by targeting adipogenesis. The antitumor mechanism of BaP needs further study, and the combination of BaP with other antitumor agents may provide a potential strategy for the treatment of AML.

## Acetyl-CoA carboxylase 1 and breast cancer

The high invasiveness and relapse rates of breast cancer are related to obesity, and obesity-related metabolic dysfunction has become the primary risk factor for postmenopausal women to develop metastatic breast cancer (Khandekar et al., 2011). ACC1 inhibition has been identified as a molecular convergence point for epithelial mesenchymal transition and tumor invasion (Rios Garcia et al., 2017). Leptin and TGFβ could inhibit ACC1 function through TGFβ-activated kinase-AMPK signaling in breast cancer. ACC1 downregulation further led to an increased level of total acetyl CoA and protein acetylation, which induced acetylation activation of Smad2 transcription factor and epithelial mesenchymal transition to enhance migrative and invasive capability of breast cancer cells (Rios Garcia et al., 2017). Specific inhibition of ACC1 in breast tumor cells impeded palmitic acid synthesis, which further induced cell apoptosis, ROS accumulation and mitochondrial dysfunction. Small molecule inhibitors targeting ACC1 could impair cell viability of breast cancer and inhibited tumor growth in xenografts derived from cell lines and patients (Chajès et al., 2006).

## Acetyl-CoA carboxylase 1 and lung cancer

Lung cancer is the leading cause of cancer-related death worldwide because of its rapid growth and high invasiveness (Sung et al., 2021). ACC1 mRNA has been found to be upregulated in non-small cell lung cancer (NSCLC), accompanied by decreased DNA methylation on the S-shore of CpG island of ACC1 (Li et al., 2019). Besides, upregulated ACC1 level was associated with poor prognosis in NSCLC patients (Li et al., 2019). Long non-coding RNAs (LncRNAs) interact with proteins to participate in the regulation of protein function. A novel lncRNA, CTD-2245E15.3, enhanced lung tumor initiation and development by regulating ACC1 (Wang et al., 2021). CTD-245E15-3 exerted its oncogenic effect by binding to ACC1 to regulate fatty acid biosynthesis in tumor cells. Knockdown of CTD-2245E15.3 increased phosphorylation of ACC1, leading to inhibition of enzyme activity. ND-646, an allosteric inhibitor of ACC1, prevented the dimerization of ACC1 subunits to block fatty acid synthesis and thus impede lung tumor growth (Li et al., 2019). ND-646 significantly inhibited lung tumor growth in KRAS p53 and KRAS Lkb1 mouse models of NSCLC (Svensson et al., 2016).

## Acetyl-CoA carboxylase 1 and liver cancer

Liver cancer leads to nearly 750,000 deaths every year, most of which are hepatocellular carcinoma (HCC) (Sung et al., 2021). ACC1 converts acetyl CoA to malonyl CoA, and malonyl CoA is the first substrate used for *de novo* lipogenesis (DNL). The increase of liver DNL is the key factor of HCC initiation (Liu and Dong, 2021). Fructose consumption and gene activation of ACC1 increase DNL and hepatocarcinogenesis. ND-654, a liver-specific ACC inhibitor, mimics the effect of ACC1 phosphorylation and impairs the progression of hepatic DNL and HCC (Lally et al., 2019). Single or in combination with sorafenib, a multi-kinase inhibitor, could improve the survival rate of tumor-bearing rats. Gene alteration led to the decrease of ACC phosphorylation level, which can promote the development of HCC cells. Although mutations in AMPK or ACC1 have not been described in HCC, there is sufficient literature to prove that obesity and type 2 diabetes, two major risk factors for HCC, are related to reduced AMPK activity and ACC phosphorylation level. These findings indicate that therapies that use small molecules like ND-654 to simulate the effect of AMPK phosphorylation on ACC may be valuable for the treatment of HCC.

## Acetyl-CoA carboxylase 1 and other malignancy

Lipidomic profiling of specimens from prostate tumor has indicated promising therapeutic targets involved in tumorigenesis of prostate cancer. Targeting lipid features by inhibiting ACC1 led to impaired tumor proliferation and increased cell apoptosis in patient-derived explants (Butler et al., 2021). ACC1 inhibition could impair tumor formation in colitis-associated colon cancer (Li et al., 2022). Blocking fatty acid synthesis by ACC1 inhibition in intestinal epithelial cells led to deceased epithelial crypt structures and reduced Lgr5+ intestinal epithelial stem cells by impairing the nuclear accumulation of PPARδ/β-catenin (Li et al., 2022). ACC1 also participates in the rewiring of tumor metabolism in response to cetuximab treatment of head and neck squamous cell carcinoma (HNSCC). HNSCC cells with acquired cetuximab resistance display elevated expression of T172-phosphorylated AMPK, S79-phosphorylated ACC1, and ACC1 (Luo et al., 2017). ACC1 rewires HNSCC metabolism from glycolysis to lipogenesis. Cetuximab plus TOFA (an allosteric inhibitor of ACC) exhibited significantly inhibitive effect on the growth of cetuximab-resistant HNSCC xenografts (Luo et al., 2017).

## Acetyl-CoA carboxylase 1 and non-tumor diseases

ACC1 is a key regulator of fatty acid metabolism, making it promising therapeutic targets for multiple metabolic diseases. ACC1 has been regarded as attractive therapeutic targets for non-alcoholic steatohepatitis (NASH). However, the use of ACC1 inhibitors tested in clinical trials is limited by side effects such as hypertriglyceridemia (Kim et al., 2017; Goedeke et al., 2018). The anti-NASH effect of ACC1 inhibitors alone or in combination with ACC1 inhibitors (PF-05221304) and diacylglycerol acyltransferase 2 (DGAT2) inhibitors (PF-06865571) has been tested in a clinical trial (Calle et al., 2021). The results showed that ACC1 inhibitor alone had obvious inhibitory effect on NASH, but it could induce hyperlipidemia, while the combination with DGAT2 inhibitor could reduce the side effects. Therefore, the combination of PF-05221304 and PF-06865571 is a potential treatment strategy, which can eliminate the side effects of PF-05221304 single treatment and bring greater clinical benefits than PF-06865571 or PF-05221304 single treatment (Calle et al., 2021). Novel ACC1-targeted anti-NASH therapeutic strategies need to be explored to successfully address the adverse effects of hyperlipidemia while maintaining the anti-NASH efficacy. IMA-1 is a small molecule for the treatment of NASH by interrupting the interaction of arachidonic acid 12 lipoxygenase (ALOX12)-ACC1 (Zhang et al., 2021). The anti-NASH effect of IMA-1 mainly depends on its direct binding to the ALOX12 pocket near the interactive surface of ACC1, rather than inhibit the activity of ALOX12 lipoxygenase. IMA-1 treatment exhibits little side effect of inhibiting ACC1 enzyme activity. These findings have brought promising results to the indications of ACC1 inhibition in anti-NASH treatment.

Fatty acid synthesis is also critical for platelet activation (Yoshida et al., 1992; Kelly et al., 2020). In the double knock-in (DKI) mouse model, Ser79 on ACC1 and Ser212 on ACC2, the phosphorylation site of AMPK, were mutated to block AMPK effect on ACC (Lepropre et al., 2018). Blood collected from ACC DKI mice showed an increase in thrombosis induced by vascular injury and growth of thrombus on the collagen coated surface. In collagen-stimulated platelets, the loss of AMPK-ACC1 signal is related to the expansion of thromboxane production and the secretion of dense granules. In general, AMPK-ACC signaling pathway controls thrombosis formation by specifically regulating the response of thromboxane and particle release to collagen. ACC inhibitor CP640.186 has been proved to increase tubulin acetylation and impair thrombin-mediated platelet aggregation (Octave et al., 2021).

## The clinical implications of targeting acetyl-CoA carboxylases

Tumor metabolic remodeling is often accompanied by changes in the expression of metabolic enzymes (Jiang et al., 2021). In addition to catalyzing intracellular metabolic reactions, metabolic enzymes also participate in the regulation of gene expression, cell cycle, DNA damage repair, cellular antioxidant capacity, proliferation, survival, apoptosis and tumor microenvironment, which further expands the metabolic dependence of tumor cells and endowing tumor cells with the ability to adapt to different environmental stimuli (Pan et al., 2021). In a study using mRNA expression data from the Cancer Genome Atlas, the expression patterns of a list of enzymes in multiple cancer types and matched normal tissues were detected to identify enzymes that were dominant in tumorigenesis. A prioritized list of new metabolic targets was developed for these cancer types with ACC1 being the most widely shared and functionally confirmed target (Marczyk et al., 2022). The expression levels of ACC1 have been analyzed by GEPIA database (Tang et al., 2017).

Soraphen A is a macrocyclic polyketide from myxomycetes, exhibiting anti-fungal activity due to its ability to bind to the dimeric interface of the BC domain of eukaryotic ACC and thereby impairing enzymatic oligomerization and blocking its activity. At nanomolar concentrations, sorafen A inhibits adipogenesis and promotes fatty acid oxidation in prostate tumor cells, while it has no cytotoxic effect on precancerous cells (Beckers et al., 2007). 5-tetracepoxy-2-furan acid (TOFA), a structural inhibitor of ACC1, exerts cytotoxic effects on lung and colon tumor cells. RNAi technology targeting ACC1 resulted in inhibition of LNCaP prostate tumor cell proliferation and caspase-induced apoptosis of highly lipogenic tumor cells (Brusselmans et al., 2005). ND-646 functions as an allosteric inhibitor of ACC1 and ACC2 by preventing ACC subunit dimerization, thus blocking fatty acid synthesis *in vitro* and *in vivo*. In several models of NSCLC, ND-646 treatment impeded fatty acid synthesis and lipid accumulation, leading to impaired tumor proliferation and enhanced cell apoptosis (Svensson et al., 2016). Collectively, there is increasing interest in the use of ACC inhibitors for anti-tumor strategies. Further evaluations of potency, pharmacokinetics, and doses of these inhibitors are required.

## Conclusion

ACC1 plays an essential role in fatty acid synthesis. For many years, the key regulatory role of ACC1 in fatty acid metabolism makes it attractive therapeutic targets for various metabolic diseases including non-alcoholic fatty liver disease, obesity and diabetes. Tumors have a high energy flow and a strong dependence on fatty acid synthesis. ACC1 inhibition has become an appealing choice for anti-tumor therapy. Some encouraging results indicated that at least some specific tumor types may respond to ACC1 inhibition. It may be one of the hot spots in future study to expand the clinical indications of ACC1 inhibitors. It is desirable to explore effective inhibitors targeting ACC1.
